# Expression Profiling Analysis of the SWEET Gene Family in In Vitro Pitaya Under Low-Temperature Stress and Study of Its Cold Resistance Mechanism

**DOI:** 10.3390/plants13213092

**Published:** 2024-11-02

**Authors:** Youjie Liu, Hanyao Zhang, Ke Zhao, Xiuqing Wei, Liang Li, Yajun Tang, Yueming Xiong, Jiahui Xu

**Affiliations:** 1 Fruit Science Institute, Fujian Academy of Agricultural Sciences, Fuzhou 350013, China; liuyoujie@faas.cn (Y.L.); weixiuqing@faas.cn (X.W.); yajun_t@163.com (Y.T.); xiongyueming@faas.cn (Y.X.); 2 Key Laboratory of Biodiversity Conservation in Southwest China, National Forest and Grassland Administration, Southwest Forestry University, Kunming 650224, China; zhanghanyao@swfu.edu.cn (H.Z.); zhaoke@swfu.edu.cn (K.Z.)

**Keywords:** *Hylocereus undatus*, SWEET gene family, low-temperature stress, response, physiological changes, molecular mechanism

## Abstract

Pitaya (*Hylocereus undatus*) fruit is an attractive, nutrient-rich tropical fruit with commercial value. However, low-temperature stress severely affects the yield and quality of pitaya. The relevant mechanisms involved in the response of pitaya to low-temperature stress remain unclear. To study whether the SWEET gene family mediates the response of *H. undatus* to low-temperature stress and the related mechanisms, we performed genome-wide identification of the SWEET gene family in pitaya, and we used ‘Baiyulong’ tissue-cultured plantlets as material in the present study. We identified 28 members of the SWEET gene family from the *H. undatus* genome and divided these family members into four groups. Members of this gene family presented some differences in the sequences of introns and exons, but the gene structure, especially the motifs, presented relatively conserved characteristics. The promoter regions of most *HuSWEETs* have multiple stress- or hormone-related cis-elements. Three duplicated gene pairs were identified, including one tandem duplication gene and two fragment duplication gene pairs. The results revealed that the *SWEET* genes may regulate the transport and distribution of soluble sugars in plants; indirectly regulate the enzyme activities of CAT, POD, and T-SOD through its expression products; and are involved in the response of pitaya to low-temperature stress and play vital roles in this process. After ABA and MeJA treatment, the expression of *HuSWEETs* changed significantly, and the cold stress was also alleviated. This study elucidated the molecular mechanism and physiological changes in the *SWEET* gene in sugar metabolism and distribution of pitaya when it experiences low-temperature stress and provided a theoretical basis for cold-resistant pitaya variety breeding.

## 1. Introduction

Pitaya fruit (*Hylocereus undatus*) is a member of the cacti family and has attracted worldwide attention because of its attractiveness and high nutritional and commercial value [1,2]. White-flesh and red-flesh pitaya fruit have been produced commercially on a large scale [3]. Hylocereus used to grow in subtropical and tropical regions of America. Currently, pitaya is grown mainly in Colombia, Mexico, Costa Rica, Nicaragua, and Vietnam [4,5]. Pitaya plants have also attracted the interest of the horticultural community because they are highly drought-resistant, allowing pitaya to grow in arid areas [6,7]. The pitaya tree is a typical tropical crop. The most suitable growth temperature is approximately 30 °C. However, it is not tolerant to low-temperature stress and freezing and can tolerate a minimum low temperature of about 5 °C. Many studies have shown that low-temperature stress can affect the yield and quality of pitaya [8]. With the further promotion of pitaya cultivation, low-temperature stress has become a vital factor for its industrial development.

Sugars are not only the prime source of carbon and energy for eukaryotes and prokaryotes but also crucial signaling molecules in various physiological processes [9]. In addition, sugar is a prime determinant of fruit quality and flavor [10]. In plants, sugar is the main product of photosynthesis, which mainly occurs in the stromal cells of chloroplasts, and it is transferred to sink organs through long-distance transport [9]. However, the movement of sugar between tissues through the phloem requires the help of sugar transporters [11]. To date, various sugar transporters have been identified in plants, and they can be divided into three families: monosaccharide transporter-like (MST), sucrose transporters (SUTs/SUCs), and sugars will eventually be exported transporters (SWEETs). The SWEET family is a family of carbohydrate transporters found in plants and mammals [11] and is characterized by the MtN3/saliva motif (PF03083) and seven transmembrane helices (TMHs) [12]. Eukaryotic SWEET has a 3-1-3 TMH structure, organized as a tandem repeat of two 3-TMH domains separated by a single transmembrane domain [13,14,15].

To date, genome-wide analysis of the *SWEET* genes has been comprehensively performed in several plant species, such as *A. thaliana* [11], rice [15], litchi [16], and pomegranate [17]. Biochemical and functional analyses revealed that the *SWEET* genes are involved in many different functions, such as phloem loading [11], nectar secretion [18], pollen development [19], erythrocyte response [20], senescence [12,21,22,23,24,25,26,27], host–pathogen interactions [22,28,29,30], and seed and fruit development [29,31,32]. *SWEET* genes play vital roles in plant resistance to low-temperature stress. *SWEET* genes are involved in plant adaptability to low-temperature environments by regulating the transport and distribution of soluble sugars in plants.

Gene family expression profiling is a method used to elucidate biological functions and regulatory mechanisms of a specific gene family in plants by studying the expression changes under different environmental conditions [33]. In the study of plant cold resistance mechanisms, by comparing the expression patterns of gene families in plants under normal and low-temperature conditions, scientists can identify genes that are significantly upregulated or downregulated in low-temperature environments and thus reveal their roles in the process of cold resistance in plants [34]. For example, some genes may be involved in the transduction of cold response signals in plants or regulate the synthesis of substances related to frost resistance. These findings can further explore how plants adapt to cold environments through gene regulation, thus providing a theoretical basis and molecular tools for improving plant cold tolerance [34]. In the study of pitaya, the analysis of the expression patterns of the SWEET gene family members in response to low temperature can reveal the molecular mechanisms of the *SWEET* genes when pitaya encounters low-temperature stress and provide some suggestions and theoretical basis for the breeding of cold-resistant pitaya varieties.

To study the mechanism of pitaya *SWEET* genes in cold resistance and the physiological changes of in vitro pitaya after being subjected to low-temperature stress, the following works were performed. This study: (1) identified 28 *SWEET* genes in pitaya and analyzed their phylogenetic relationships, gene structures, protein motifs, and locations on the chromosome; (2) analyzed relevant physiological indexes, including soluble sugar content, CAT, T-SOD, and POD enzyme activities, and growth condition after treating with low-temperatures; and (3) analyzed the expression profiles of *SWEET* genes under low-temperature stress and the application of exogenous ABA and MeJA.

## 2. Results

### 2.1. Identification of the HuSWEET Gene Family

A total of 28 nonredundant *HuSWEETs* were detected in the pitaya genome. The number of amino acids, molecular weight (MW), and isoelectric point (pI) were calculated based on the protein sequence of each identified SWEET. The number of predicted amino acids in HuSWEET was 135 (HuSWEET13) to 335 (HuSWEET17). The range was 14,964.35 (HuSWEET13)–37,596.06 kDa (HuSWEET6). The theoretical pI value was 5.71 (HuSWEET6)–9.69 (HuSWEET29). The hydrophilicity (GRAVY) value was in the range of 0.152 (HuSWEET9) to 0.939 (HuSWEET13), indicating that it is hydrophobic. With the hydrophobic characteristics of transmembrane proteins, out of 28 HuSWEETs, HuSWEET20 had seven THMs, and HuSWEET8 had the fewest THMs, with only four. Subcellular localization analysis revealed that HuSWEET was predicted to be located at the cell membrane (Table 1), which was consistent with previous findings that most SWEET members in *A. thaliana* and rice are also transmembrane proteins on the cell membrane [11,15] whose prime function may be the transport of soluble sugars, including sucrose and maltose.

### 2.2. Phylogenetic Analysis of the HuSWEET Genes

**Figure 1:**
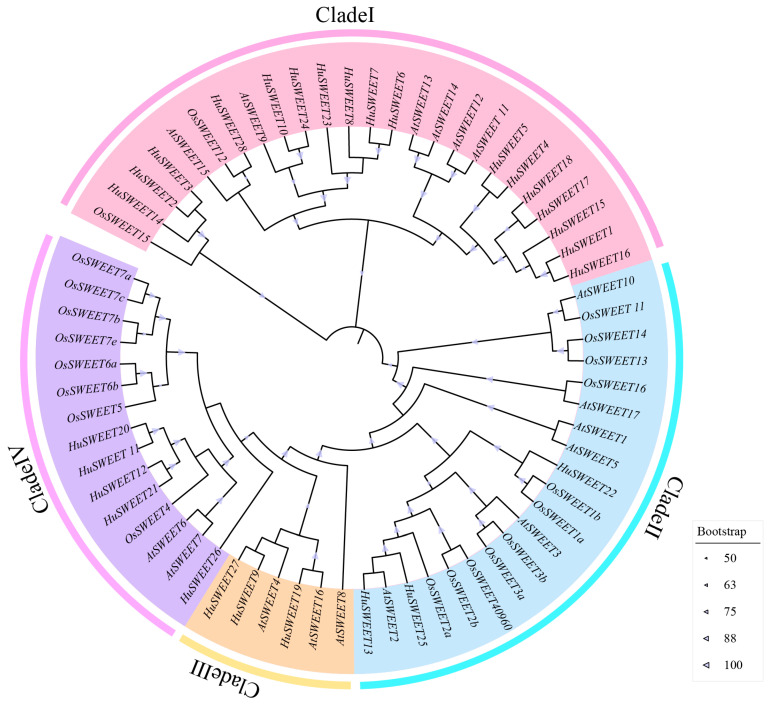
Phylogenetic tree of *SWEET* sequences from *Hylocereus undatus*, *Arabidopsis thaliana*, and *Oryza sativa*. Note: subfamilies I, II, III, and IV are represented in pink, light blue, light orange, and purple, respectively. The phylogenetic tree was constructed using the maximum likelihood method, and 1000 repeats were used. Black means that in 1000 repeats, bootstrap values > 90; gray means that in 1000 repeats, 90 > bootstrap values > 70; and white gray means values < 70.

As shown in Figure 1, all members of the *HuSWEET* gene were divided into four subfamilies, which was essentially consistent with the grouping of *A. thaliana* (divided into four subfamilies) but different from the grouping of rice (divided into three subfamilies) slightly. Among them, subfamily I was the largest clade and contained 17 *HuSWEET* members, six *AtSWEET* members, and two *OsSWEET* members; subfamily II included three *HuSWEET* members, six *AtSWEET* members, and 11 *OsSWEET* members; subfamily III consisted of three *HuSWEET genes* and three *AtSWEET* genes and subfamily IV contained five *HUSWEET* genes, two *AtSWEET* genes, and eight *OsSWEET* genes. These findings indicated that the phylogeny of the SWEET gene family in pitaya was closer to that of the dicot *A. thaliana* and significantly different from that of the monocot rice.

### 2.3. Conserved Motif and Gene Structure Analysis

**Figure 2:**
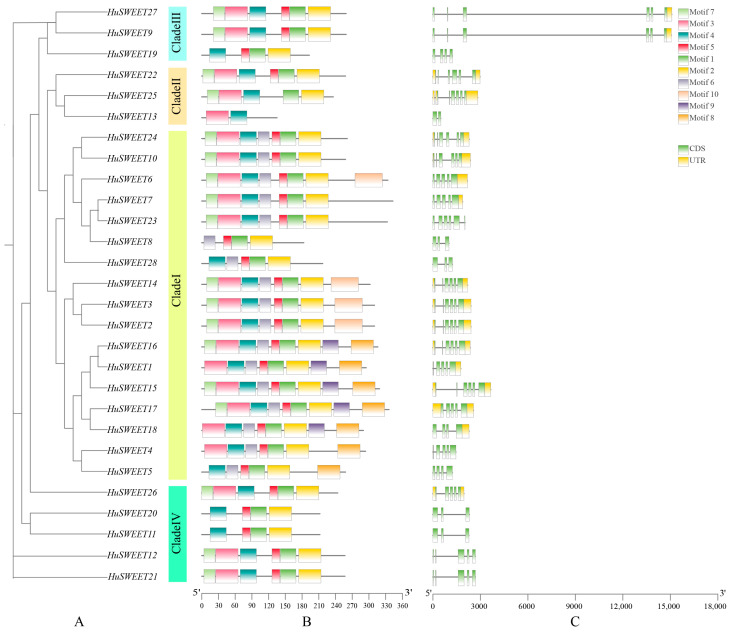
Analysis of conserved motifs and gene structure of *HuSWEET* genes. Note: (**A**) Members of the HuSWEET gene family. (**B**) Motif composition of HuSWEET protein. Ten patterns are displayed in rectangles of different colors. (**C**) The green, yellow, and black exon–intron groups of the *HuSWEET* gene correspond to the non-coding region, exon, and intron, respectively.

To explore the sequence characteristics of the HuSWEET proteins, MEME software and TBtools were used to predict and map the conserved domains. The results revealed ten different motifs in the SWEET protein family. These motifs are displayed in Figure 2A. They represent the typical pattern of PmSWEET protein motifs. For example, the HuSWEET8 protein contained only two motifs, and the other 27 HuSWEET proteins contained at least four. Motifs 1, 2, 3, 4, and 6 presented a high degree of conservation, consistent with the conserved domains reported by Wen et al. [35]. This discovery not only confirmed the SWEET protein family conservation during the evolutionary process but also provided important clues for our understanding of the functions and mechanisms of these proteins.

Structures were further analyzed to elucidate the structural characteristics of the HuSWEETs. The results (Figure 2B) revealed that the gene structure of most of the HuSWEET gene family genes was conserved. The 28 *HuSWEET* genes contained two to six exons and one to six introns, of which three (*HuSWEET8* and *HuSWEET28*) to six (*HuSWEET2, 3, 10, 14, 15, 16*) subfamily I members were involved in exons and two (HuSWEET8 and HuSWEET28) to five (*HuSWEET2, 3, 10, 14, 15, 16*) introns; subfamily II had two (*HuSWEET13*) to five (*HuSWEET22* and *HuSWEET25*) exons and one (*HuSWEET13*) to six (*HuSWEET25*) introns; and subfamily III had four (*HuSWEET19*) to six (*HuSWEET9* and *HuSWEET27*) exons and three (*HuSWEET19*) to five (*HuSWEET9* and *HuSWEET27*) introns. Subfamily IV had three (*HuSWEET11* and *HuSWEET20*) to five (*HuSWEET12*, *HuSWEET21,* and *HuSWEET26*) exons and two (*HuSWEET11* and *HuSWEET20*) to four (*HuSWEET12*, *HuSWEET21* and *HuSWEET26*) introns. Even members of the same subfamily have certain differences in their gene structure. For example, the gene structures of *HuSWEET13* and *HuSWEET19* differ greatly from those of other genes in the subfamily to which they belong. It is speculated that *HuSWEET13* and *HuSWEET19* may have undergone unique evolution. Although these gene family members present some differences in the sequences of introns and exons, the gene structure, especially the motifs, is relatively conserved. These results indicate that the members of this gene family may have some functional conservation (e.g., transport of soluble sugars) and diversification, e.g., participation in the soluble sugars’ transport under different abiotic stresses.

### 2.4. Cis-Acting Element Analysis of HuSWEET Gene Family Members

**Figure 3:**
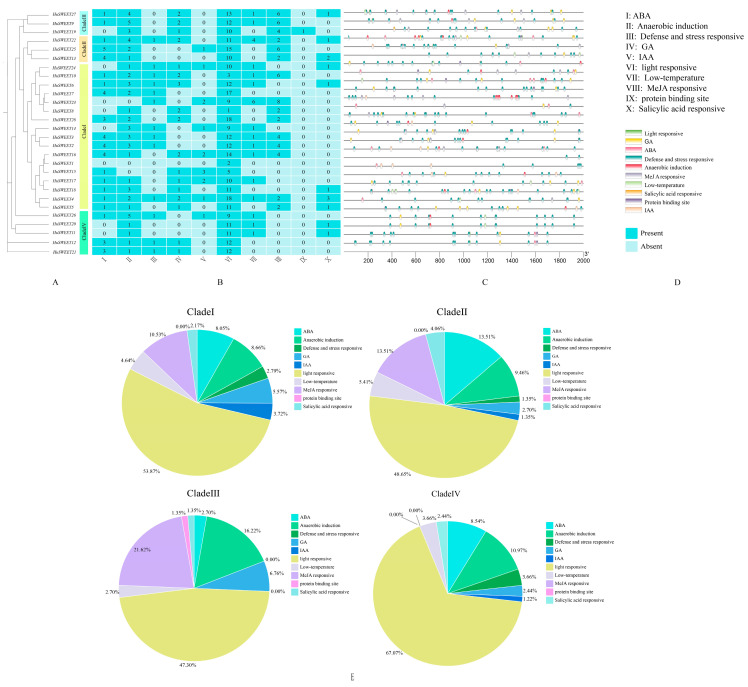
Distribution of cis-acting elements in the 2000 bp sequence upstream of the *HuSWEET* gene. Note: different cis-acting elements are represented by different colors. (**A**) members of the HuSWEET gene family; (**A**) the number of cis-acting elements of each *HuSWEET* gene; (**B**) the numbers in the heatmap box indicate the number of different elements in these HuSWEET; (**C**) the position of each cis-acting element; (**D**) class of cis-acting element; (**E**) percentage of *HuSWEET* gene promoter cis-elements in different subfamilies, with different colors representing different promoter cis-element classifications.

Generally, 2000 bp upstream of the transcription start site was chosen to be studied as the promoter region by default because the upstream region of a gene usually contains the regulatory elements of the gene, generally within 2000 bp of the gene’s action [36]. The cis-acting elements of the 2000 bp upstream sequences of the 28 genes were analyzed via PlantCARE and TBtools. The results revealed that, except for *HuSWEET1*, the promoter regions of the remaining *HuSWEETs* presented multiple stress- or hormone-related cis-elements (Figure 3A–D), in which light responsiveness was distributed in each gene. Hormone-related cis-acting elements, such as jasmonic acid (MeJA), abscisic acid (ABA), gibberellin (GA), salicylic acid, and indole acetic acid (IAA), are second only to light-responsive elements. Figure 3E reveals that the light-responsive element had the highest proportion in all subfamilies; quantitatively, the defense and stress-responsive, GA, and IAA elements were mainly in subfamily I, and the three elements also had a high proportion in subfamily I; the ABA and MeJA elements had a higher proportion in subfamily II; subfamily III did not contain the defense and stress-responsive and IAA elements; the protein binding site was only distributed in subfamily III in *HuSWEET19*; subfamily III had the highest number and proportion of MeJA and salicylic acid elements, while subfamily IV did not contain MeJA elements; and low-temperature elements were more distributed in *HuSWEET22* of subclade II and *HuSWEET23* of subclade I, suggesting that these two genes might play a role when pitaya plants are under a cold-stress environment. These results indicate that HuSWEET proteins are associated with many biological functions and environmental responses, which may illustrate that they play crucial roles in plant growth, development, defense mechanisms, and environment adaptability.

### 2.5. Chromosomal Location, Gene Duplication, and Synteny Analysis of the HuSWEET Gene Family Members

TBtools software was used to perform mapping analysis on 11 chromosomes of the pitaya genome for 28 *HuSWEET* genes to understand the chromosome distribution and genome-wide density of pitaya *HuSWEET* genes. The results revealed that all 28 *HuSWEETs* were located on chromosomes; the *HuSWEET* genes were not distributed on chromosomes 5, 6, and 11; the *HuSWEET* genes were located mainly on chromosome 4; there were a total of 11 *HuSWEETs*; and there was only one *HuSWEET* member on chromosomes 3 and 10: *HuSWEET10* and *HuSWEET28*, respectively. In addition, most genes were located in areas with relatively high chromosome density.

**Figure 4:**
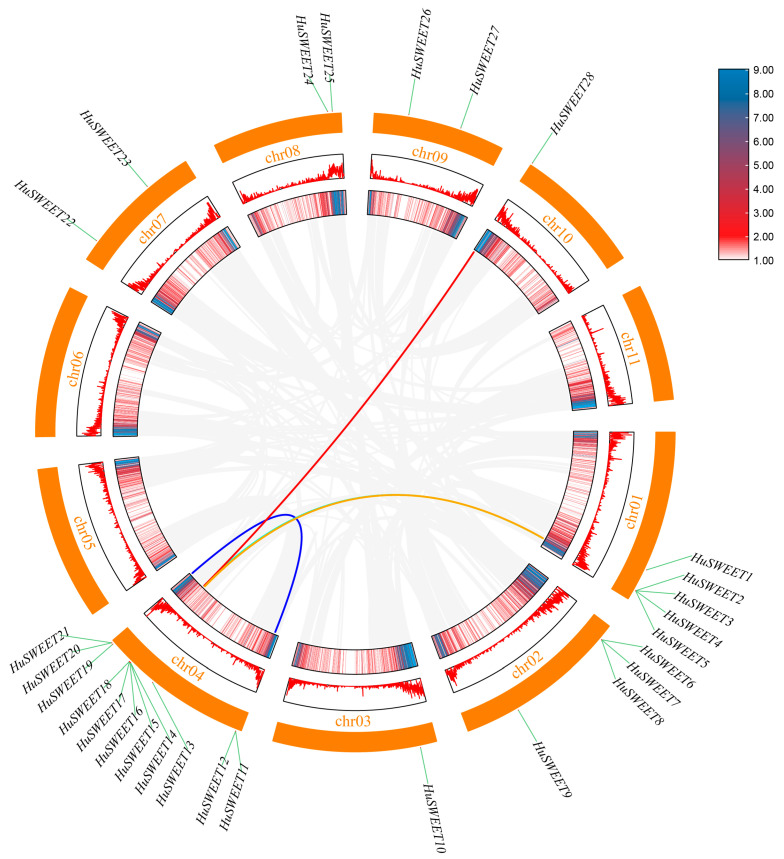
Collinearity analysis of *HuSWEET* gene in *H. undatus*. Note: gray lines represent all duplicate genes, red and orange lines represent fragments of *HuSWEET* gene pairs, and blue lines represent tandem duplicate gene pairs. The heat map and line map are gene densities and the line map density increases from white to red to blue. The yellow rectangles are chromosomes, and the chromosome names are displayed between each chromosome and the gene density.

When the evolutionary history of the SWEET gene family in the pitaya (*H. undatus*) genome was explored, gene duplication events were considered the core mechanism driving the evolution of this family. To this end, we performed an exhaustive gene replication analysis of 28 *HuSWEET* genes in pitaya (Figure 4). The results revealed significant gene segment duplications. Three duplicated gene pairs were identified, including one pair of tandem and two fragment duplication gene pairs. The tandem duplication of genes is distributed on chromosome 4, occurring at the two ends of the chromosome. Notably, chromosome 4 was a hotspot region that carried 11 AcNRAMP genes and recorded an independent gene duplication event. The duplications of these three fragments suggested that they played some roles in driving the expansion, functional differentiation, and evolution of the HuSWEET gene family.

**Figure 5:**
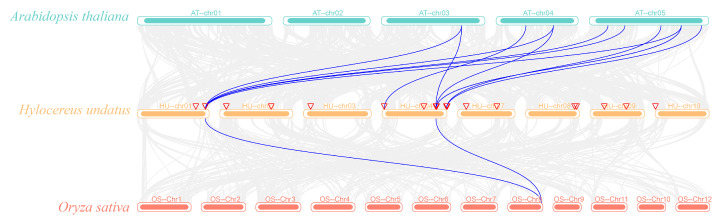
The collinear distribution of *H. undatus*, *A. thaliana*, and *O. sativa*. Note: the blue line connects the *HuSWEET* gene with the collinear relationship of *A. thaliana* and *O. sativa*. The gray lines connect the collinearity of other genes. Hu-, At-, and Os- represent the chromosomes of *H. undatus*, *A. thaliana*, and *O. sativa*, respectively, followed by chromosome serial numbers.

In addition, this study analyzed the *HuSWEET* collinear relationships among pitaya, *A. thaliana*, and rice (Figure 5). The results revealed that pitaya and rice presented two pairs of homologous genes, mainly on chromosome 8. There were 11 pairs of homologous genes associated with *A. thaliana*, and the homologous genes were located on chromosomes 1 and 4, with a high frequency of occurrence. These results suggest that, compared with that of monocots, the evolutionary relationship between dicots and pitaya is closer.

### 2.6. Changes in Relevant Indicators Before and After Low-Temperature Stress

**Figure 6:**
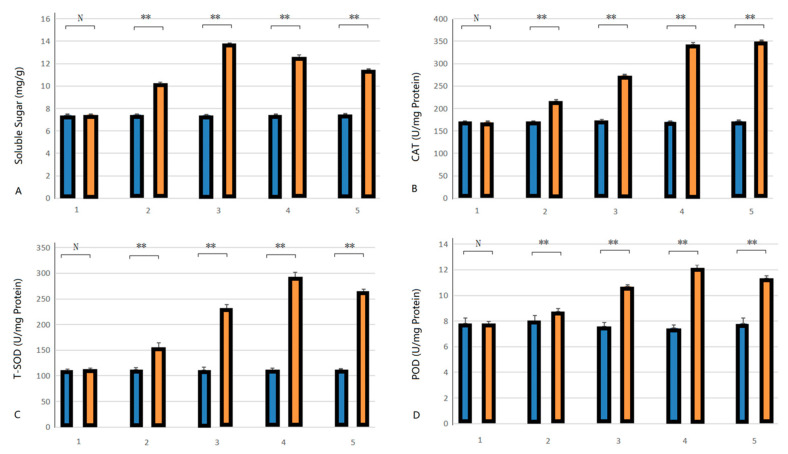
Changes in soluble sugar content, CAT, T-SOD, and POD enzyme activities in *H. undatus* after low-temperature stress treatments. Note: (**A**) soluble sugar content; (**B**) CAT enzyme activity; (**C**) T-SOD enzyme activity; (**D**) POD enzyme activity. 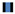, control; 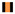, treatment. 1, 2, 3, 4, and 5 represent 0 h, 12 h, 24 h, 48 h, and 72 h, respectively, at 4 °C. **, an extremely significant difference (*p* < 0.01); and N, no significant difference (*p* > 0.05).

Scavenging excess reactive oxygen species and free radicals and protecting plant cells from oxidative damage are the primary functions of CAT, T-SOD, and POD enzymes in plants [37]. These enzymes work synergistically through different pathways and mechanisms to maintain oxidative homeostasis in plants. After in vitro pitaya seedlings were subjected to low-temperature stress, the soluble sugar content and T-SOD and POD enzyme activities all tended to increase firstly but then decrease (Figure 6A,C,D), while the CAT activity tended to increase gradually (Figure 6B). Specifically, the soluble sugar content of leaves increased by 84% within 0–24 h of low-temperature treatment; there was a downward trend from 24 to 72 h, and the soluble sugar content decreased by 12.85% during this period. CAT activity increased by 104.90% within 0–72 h. T-SOD activity increased by 54.93% within 0–48 h and decreased by 11.16% within 48–72 h. POD activity increased by 164.11% within 0–48 h and decreased by 9.17% within 48–72 h. These findings indicate that the soluble sugar content and the CAT, T-SOD, and POD enzyme activities of in vitro pitaya seedlings significantly changed after low-temperature stress.

### 2.7. Changes in the Expression Profiles of the SWEET Gene Family

**Figure 7:**
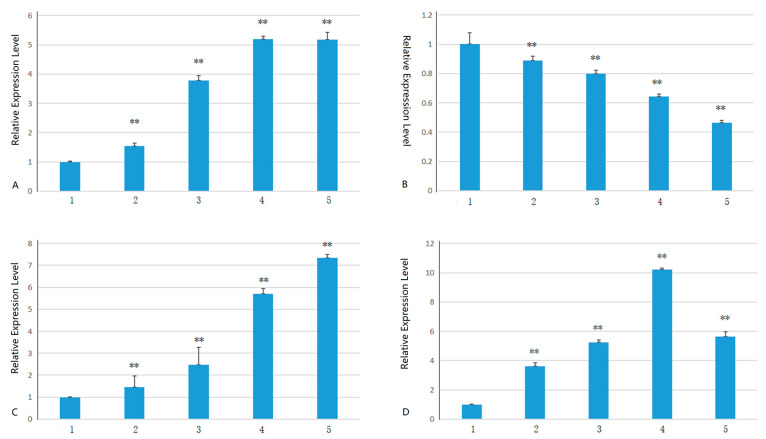
Changes in expression levels of SWEET gene family members related to pitaya’s low-temperature stress response after low-temperature stress. Notes: (**A**) *HuSWEET5*; (**B**) *HuSWEET12*; (**C**) *HuSWEET14*; (**D**) *HuSWEET21*. 1, 2, 3, 4, 5 represent 0 h, 6 h, 12 h, 48 h, 72 h, respectively, at 4 °C. **, an extremely significant difference (*p* < 0.01).

Of the 28 family members, only 14 were expressed at room temperature, and 14 were not expressed at room temperature (see Supplementary Table S1). Some genes, such as *HuSWEET28*, were expressed after cold treatment but not at room temperature; 14 genes were not expressed at room temperature. Among the genes expressed at room temperature, only four genes were significantly different from each other after cold treatment and at room temperature. However, the other ten genes were not significantly different (see Supplementary Table S1). Further analysis of the genes whose expression significantly differed revealed that the expression levels of *HuSWEET5*, *HuSWEET14*, and *HuSWEET21* increased with increasing low-temperature stress (Figure 7A,C,D). However, the expression level of *HuSWEET12* decreased with increasing low-temperature stress time (Figure 7B).

### 2.8. Effects of Exogenous ABA and MeJA on the Expression and Growth of HuSWEETs

**Figure 8:**
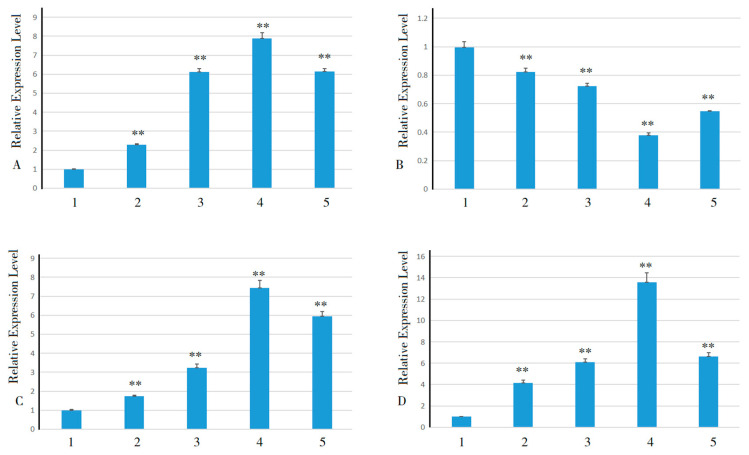
Expression changes of SWEET gene family members associated with low-temperature stress response in pitaya after application of ABA and low-temperature stress. Note: (**A**) *HuSWEET5*; (**B**) *HuSWEET12*; (**C**) *HuSWEET14*; (**D**) *HuSWEET21*. 1, 2, 3, 4, and 5 represent treatments at 4 °C for 0 h, 6 h, 12 h, 48 h, and 72 h, respectively. **, highly significant differences (*p* < 0.01).

The expression pattern of *HuSWEETs* under ABA treatment was analyzed by qRT-PCR to clarify whether the expression of *HuSWEETs* responded to ABA signaling. The results showed that *HuSWEET5*, *HuSWEET14*, and *HuSWEET21* showed a trend of upward and then downward expression under the treatment of exogenous ABA hormone (Figure 8), and *HuSWEET12* showed a trend of downward expression.

**Figure 9:**
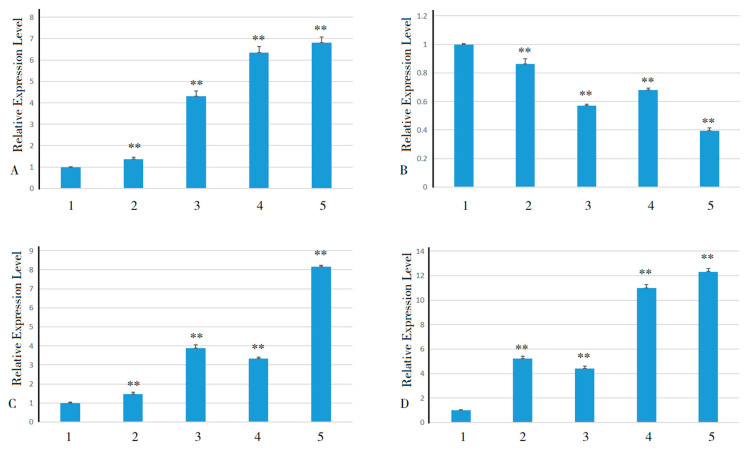
Changes in the expression of SWEET gene family members associated with low-temperature stress response in pitaya after application of MeJA subjected to low-temperature stress. Note: (**A**) *HuSWEET5*; (**B**) *HuSWEET12*; (**C**) *HuSWEET14*; (**D**) *HuSWEET21*. 1, 2, 3, 4, and 5 represent treatments at 4 °C for 0 h, 6 h, 12 h, 48 h, and 72 h, respectively. **, highly significant differences (*p* < 0.01).

Under exogenous MeJA treatment, *HuSWEET5*, *HuSWEET14*, and *HuSWEET21* showed a trend of upregulated expression (Figure 9). *HuSWEET12*, on the other hand, showed a trend of downregulated expression, which was consistent with that in the absence of any exogenous hormone and ABA application. It suggests that *HuSWEET12* may be involved in the low-temperature stress negative regulation.

**Figure 10:**
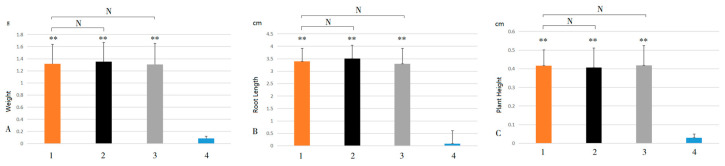
Growth condition of in vitro pitaya seedlings after being subjected to low-temperature stress. Note: (**A**) is the difference in weight; (**B**) is the difference in root length; (**C**) is the difference in plant height. Treatment 1, 30 d at room temperature; 2, 30 d at room temperature after the addition of ABA to the medium and low-temperature stress for 72 h; 3, 30 d at room temperature after the addition of MeJA to the medium and low-temperature stress for 72 h; 4, 30 d at room temperature after low-temperature stress for 72 h. **, compared with treatment 4, the difference was highly significant (*p* < 0.01); N, compared with treatment 4, the difference was not significant (*p* > 0.05).

After the low-temperature stress treatment, the seedlings were placed at room temperature for 30 d. The weight, root length, and plant height of low-temperature-stressed in vitro pitaya seedlings grew only 0.086 g, 3.398 cm, and 0.416 cm on average, whereas the control grew about 1.318 g, 0.09 cm, and 0.03 cm on average (Figure 10); the differences were highly significant (*p*-values of 0.000488875, 0.0000510821, and 0.00546807, all < 0.01). It indicates that low-temperature stress had a significant effect on pitaya seedling weight, root length, and plant height. After adding ABA or MeJA to the medium, followed by low-temperature stress, the pitaya seedling’s weight, root length, and plant height were not significantly different from those at room temperature (all *p*-values > 0.05). It indicates that adding ABA or MeJA can effectively enhance the ability of in vitro pitaya seedlings to resist low-temperature stress.

### 2.9. Three-Dimensional Structures of SWEET Gene Family Member Proteins Related to the Pitaya Low-Temperature Stress Response

**Figure 11:**
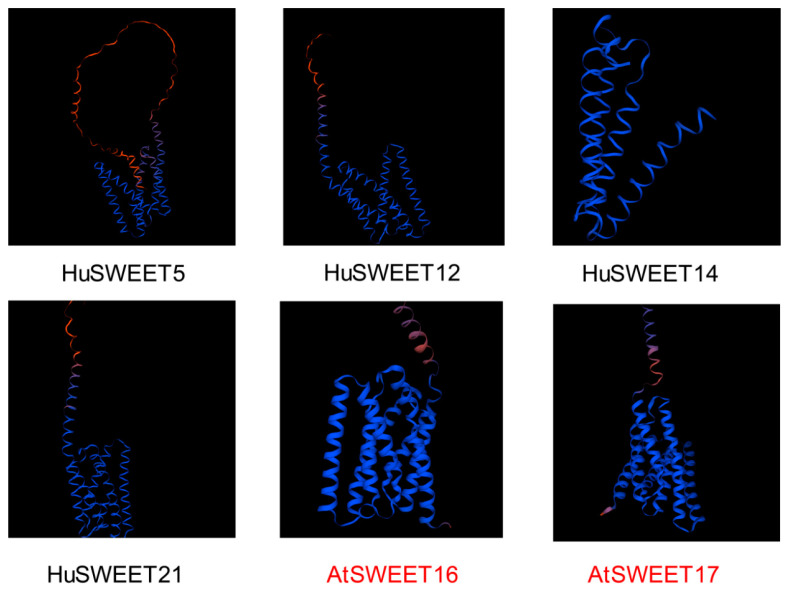
Three-dimensional structures of the SWEET gene family member proteins related to the low-temperature stress response.

The expression profiling analysis revealed that the expression levels of four genes, *HuSWEET5*, *HuSWEET12*, *HuSWEET14*, and *HuSWEET21*, significantly differed before and after low-temperature stress treatment. Therefore, these four genes are speculated to be members of the SWEET gene family associated with the low-temperature stress response in pitaya. We further predicted protein structures and found that the structural characteristics of HuSWEET5, HuSWEET12, HuSWEET14, and HuSWEET21 were similar to those of *A. thaliana* SWEET16 [38] (Figure 11), mainly beta-helix, which has been proven to play an important role in sugar transport. They have similar functions.

## 3. Discussion

Pitaya is one of the most widely planted tropical fruits with a high economic value [39]. In recent years, owing to the intensification of climate change, the development of the pitaya industry has been affected. Therefore, breeding new varieties of pitaya resistant to low-temperature stress, mining low-temperature stress genes, and studying the mechanism of low-temperature stress tolerance are vital. The SWEET gene family members play a role in plant responses to abiotic stresses, including low-temperature stress [40]. This study identified the SWEET gene family of pitaya fruit, analyzed the expression patterns of *HuSWEET* genes under low-temperature stress, and analyzed the soluble sugar content and the activities of the enzymes CAT, T-SOD, and POD under low-temperature stress to provide a reference for breeding new varieties of pitaya fruit with tolerance to low-temperature stress.

This study identified 28 *SWEET* genes in pitaya, 17 in *A. thaliana* [11], and 52 in soybean [41]. Twenty-one *SWEET* genes were identified in rice [15], and 20 genes were identified in pomegranate [12], indicating that the number of *SWEET* genes is diverse in different species. Phylogenetic analysis divided the *HuSWEET* genes into four subfamilies, consistent with the number of SWEET subfamilies in *A. thaliana* but different from that in rice. Notably, among the four candidate genes involved in the low-temperature stress response screened in this study, *HuSWEET5* and *HuSWEET14* were clustered in subfamily I, and *HuSWEET12* and *HuSWEET21* were clustered in subfamily IV. Gene structure analysis revealed that the number of exons and introns in the *SWEET* gene in pitaya ranged from two to six and one to six, respectively. There were differences in the number of exons and introns in the *SWEET* genes of pitaya, indicating that the functions of the genes of different family members may differ. In summary, the similarity between gene structure and the structural characteristics of the conserved motifs indicate that *SWEETs* may have similar functions during the evolutionary process.

Promoter cis-acting elements play vital roles in transcription and gene expression regulation. This study revealed many resistance-related cis-acting elements in the upstream 2000 bp sequence of the *SWEET* gene in pitaya. The cis-acting elements in *HuSWEET* are primely divided into five categories: light response, phytohormone response, defense and stress response, anaerobic induction, and low-temperature induction, with plant hormone response elements accounting for the highest proportion. The phytohormone response elements in the *SWEET* genes of pitaya are primely abscisic acid (ABA) response elements, gibberellin (GA) response elements, methyl jasmonate (MeJA) response elements, and salicylic acid (SA) and auxin (IAA) response elements. Studies have shown that the SWEET gene family plays a crucial role in secondary metabolism regulation, such as the signal transduction of JA, ABA, GA, and SA [42]. Previous studies have shown that both MeJA and ABA can stimulate the expression of plant defense genes and induce plant chemical defenses and some physiological stress responses [43]. Some studies have shown that changing the level and signaling of GA can affect the cold tolerance of plants [44]; SA signaling can regulate plant seed germination, growth, flower induction, thermogenesis, and biotic and abiotic stress responses [45]. Therefore, the SWEET gene family members in pitaya also play crucial roles in plant growth and development regulation and low-temperature stress. Notably, among the four low-temperature stress response candidate genes screened in this study, among the 2000 bp upstream sequences of genes, only *HuSWEET14* had low-temperature response elements, whereas *HuSWEET5*, *HuSWEET12*, *HuSWEET14*, and *HuSWEET21* all contained defense- and stress-responsive response elements. These findings indicate that HuSWEET family members may operate via different mechanisms in response to low-temperature stress.

Gene or fragment duplication is the prime method of gene amplification and evolution to generate genes with new functions [46]. This study analyzed the gene duplication events of the HuSWEET gene family in pitaya and identified three pairs of genes or fragmented gene pairs among 28 *SWEET* genes (Figure 5). These findings indicate that gene duplication events are also a vital means of *SWEET* gene amplification. In addition, the segment duplication gene pairs occurred the most frequently on chromosome 4, accounting for four of the six pairs, and more *SWEET* genes were mapped to chromosomes 1 and 4, with five and 11 genes, respectively. These results indicate chromosomes 1 and 4 may contain crucial *SWEET* gene resources.

Low-temperature stress is one of the prime environmental stresses that limits plant growth and crop yield [34]. In this study, 30 pitaya tissue-cultured plantlets were divided into two groups, with 15 plants in each group. One group was subjected to low-temperature stress at 4 °C for 72 h and then grown at room temperature for 30 d. Under low-temperature stress, the tips of the pitaya tissue-cultured seedlings turned white and vitrified; compared with those in the control group, the growth amount significantly decreased, and the growth vigor was poor (Figure 10). This change indicates that low-temperature stress can limit the nutrient uptake of pitaya and inhibit plant growth [34].

The SWEET (sugars will eventually be exported transporter) gene family is a novel sugar transporter gene family. They regulate the partitioning process of carbohydrates, maintain the balance of hexose and sucrose in plants, and play a key role in plant growth and development and physiological metabolism [47]. SWEET proteins provide bidirectional transmembrane transport of sugars along the concentration gradient and play crucial roles in plant phloem loading of photosynthetic compounds, nectar secretion from nectary glands, seed filling, pollen development, pathogen interaction, and stress regulation [48]. SWEET proteins generally contain seven transmembrane domains (TMs), among which the fourth TM is less conserved and mainly plays a linking role. The protein is divided into two MtN3/saliva domains, each containing three TMs, forming 3-1-3 structures [48].

*SWEET* genes play crucial roles in plant cold resistance. The topology of SWEET proteins is significantly different from that of traditional sugar transporters (such as MSTs and SUTs), which may be a vital reason for the ability of SWEET proteins to transport sugar from inside to outside of the cell [47]. SWEET gene family members participate in the adaptability of plants to low-temperature stress by regulating the transport and distribution of soluble sugars in plants [47]. Under low-temperature stress, plants respond to stress by adjusting their metabolic pathways, in which the accumulation of soluble sugars is vital. Under low-temperature stress, the content of soluble sugars (such as sucrose, glucose, and fructose) in plants usually increases to serve as osmotic regulatory substances to help maintain the osmotic potential balance of cells and protect the cell membrane from low-temperature damage. Jiang et al. (2023) found two HuSWEET genes showed strong preferential expressions in fruits and an increase during fruit maturation, illustrating they play crucial roles in sugar accumulation in pitaya [49]. In this study, under low-temperature stress, the expression levels of some *SWEET* genes (such as *HuSWEET5, HuSWEET12, HuSWEET14*, and *HuSWEET21*) changed significantly, and the soluble sugar content also increased significantly (*p* < 0.01), thus affecting the cold tolerance of plants. *SWEET* genes affect plant adaptability to low temperatures by regulating the transport and distribution of soluble sugars (Figure 9). The change in soluble sugars in plants after low-temperature stress is a crucial aspect of the plant physiology study [50]. These changes are closely related to plant cold tolerance, dormancy release, and growth and development. During low-temperature stress, the soluble sugar content of plants usually changes significantly. These changes may manifest as an increase or a decrease in soluble sugar content, depending on the plant species, low-temperature stress treatment conditions, duration of low-temperature stress treatment, and type of soluble sugars [50]. In general, low-temperature treatment can promote sucrose accumulation in plants, but long-term or extremely low-temperature treatment can cause a decrease in sucrose content. In this study, during low-temperature treatment, the soluble sugar content of in vitro pitaya seedlings first increased but then decreased. Soluble sugars are important energy substances in plants, and their changes under cold treatment have vital effects on cold hardness, growth, and development [47]. These sugars provide energy support to plants through pathways such as glycolysis and participate in the plant’s response to adverse stress through the sugar signaling pathway [47].

Through the transport regulation and distribution of soluble sugars, SWEET gene family members indirectly affect the activity of antioxidant enzymes and the antioxidant capacity of plants (Figure 10). Under low-temperature stress, changes in the activities of CAT, SOD, and POD are vital physiological responses of plants to address low-temperature stress, and their activities usually tend to increase [12,51,52]. These studies indicated that the increase in the activities of CAT, SOD, and POD was a general adaptive response of plants to low-temperature stress. In this study, the activities of the enzymes CAT, T-SOD, and POD significantly changed after the plants were exposed to low-temperature stress. Low-temperature stress causes plants to produce large amounts of reactive oxygen species (ROS), which cause oxidative damage to cells. In addition, antioxidant enzymes (such as SOD and POD) can remove these reactive oxygen species and protect cells from oxidative damage [51]. Although *SWEET* genes are not directly involved in the synthesis regulation or activity of antioxidant enzymes, they can indirectly affect the activity of antioxidant enzymes and the antioxidant capacity of plants.

*SWEET* genes may also indirectly affect the cold tolerance of plants by affecting indicators of plant photosynthesis and respiration (such as the photosynthetic rate, respiration rate, and chlorophyll content) [53]. For example, by regulating the transport and distribution of sugar, the *SWEET* genes may help maintain normal photosynthesis and improve the energy supply and substance synthesis ability at low temperatures [25]. Notably, the above physiological indicators do not exist in isolation. These genes are interrelated and affect each other to form a complex network of plant cold resistance genes. Moreover, different plant species and cultivars may have different cold tolerances; therefore, the specific action mechanisms of the *SWEET* genes in various plants may also differ.

The exogenous hormones ABA and MeJA play vital roles in plant cold stress response [54,55]. ABA, as a crucial phytohormone, enhances plant resistance to cold stress, and it increases freezing tolerance in plants by promoting the expression of cold-responsive genes [54]. In addition, ABA is involved in regulating the antioxidant system in plants, reducing the accumulation of reactive oxygen species, and protecting cells from cold damage. MeJA, as a jasmonic acid-like hormone, is also involved in plant response to cold stress [55]. Studies have shown that exogenous MeJA treatment induces cold tolerance in banana fruits and enhances the cold tolerance of plants by interacting with transcription factors and regulating the expression of related genes [56]. The results of this study indicate that exogenous ABA and MeJA can participate in the response and adaptation mechanisms of pitaya to cold stress through different signaling pathways and interactions, which can help to improve the resistance of pitaya to low-temperature stress.

RT-PCR can detect and analyze changes in the expression levels of SWEET gene family members under cold treatment conditions, which provides vital information for elucidating the mechanism of plant cold resistance. The change in the gene family expression profile under cold treatment is one of the crucial mechanisms for plant adaptation to low-temperature stress. Different plant species, diverse gene families, and specific cold treatment conditions may lead to significant differences in gene expression profiles. With the continuous development of molecular biology techniques, we can elucidate the regulatory mechanisms of gene expression in plants under cold treatment in greater depth. Although there are no reports on using the *SWEET* gene to improve the cold resistance of pitaya, existing studies have shown that the *SWEET* gene can play a similar role in other plants. For example, in corn, the expression level of the *ZmSWEET15a* gene significantly increased under low-temperature stress, and the sucrose content in the transgenic plants significantly decreased. These findings indicate that *ZmSWEET15a* plays a crucial role in the low-temperature stress response. The overexpression of *ZmSWEET15a* can improve the cold resistance of corn. Similarly, it can be speculated that similar *SWEET* genes also exist in pitaya. These genes participate in the adaptability of pitaya to low-temperature stress by regulating the transport and distribution of soluble sugars, thereby improving the cold resistance of pitaya.

In summary, the *SWEET* genes are involved in various physiological processes in pitaya plants, including the response process to low-temperature stress, thereby improving the cold resistance of pitaya by regulating the transport and distribution of soluble sugars in plants. However, the specific functions of related genes, such as *HuSWEET5, HuSWEET12, HuSWEET14*, and *HuSWEET21*, still need further in-depth study.

## 4. Materials and Methods

### 4.1. Data Acquisition

To explore the phylogeny of *SWEET* genes in pitaya (*H*. *undatus*) and other species, we downloaded the protein sequence of pitaya from the pitaya genome database (http://www.pitayagenomic.com/ (accessed on 5 May 2023)) and the protein sequence from the Pfam database (http://pfam.xfam.org/ (accessed on 22 May 2023)). We downloaded the hidden Markov model (HMM) map of the MtN03083_slv domain of the SWEET gene family (PF03083). We obtained the TAIR database from the Arabidopsis thaliana genome website (http://www.arabidopsis.org/ (accessed on 22 May 2023)) and downloaded the protein sequences of 17 *AtSWEETs* and the protein sequences of 21 *OsSWEETs* from the rice genome website TIRG (http://rice.plantbiology.msu.edu/ (accessed on 22 May 2023)).

### 4.2. Identification and Analysis of the Physicochemical Properties and Subcellular Localization of the SWEET Gene Family in Pitaya

Based on the data downloaded above, HMMER software (version 3.1b2, http://hmmer.org/ (accessed on 22 May 2023)) was used to search the conserved domain of the SWEET gene family MtN3_slv in the pitaya protein database (ID: PF03083) [57]. The E value cutoff was set to 10^−5^ to ensure confidence [58]. Then, through SMART (http://smart.embl-heidelberg.de/ (accessed on 27 May 2023)), the Pfam database (http://pfam.xfam.org/ (accessed on 27 May 2023)) and NCBI-CDD (https://www.ncbi.nlm.nih.gov/cdd (accessed on 21 June 2023)) were used to screen all putative SWEET proteins to confirm the existence of the MtN3_slv domain and retain the sequences with the MtN3_slv domain.

The *SWEET* genes were named based on their positional information in the *H. undatus* genome. Additionally, the online ExPASy program (https://www.expasy.org/ (accessed on 21 June 2023)) was used to calculate the number of amino acids, molecular weight (MW), and isoelectric point (pI) [59]. The distribution of transmembrane helices (TMHs) was analyzed via the TMHMM server v.2.0 (https://services.healthtech.dtu.dk/service.php?TMHMM-2.0 (accessed on 21 June 2023)) for prediction [59]. The subcellular location prediction was performed via Plant-mPLoc (Plant-mPLoc server (sjtu.edu.cn)) online tool (version 2.0); the website address is http://www.csbio.sjtu.edu.cn/bioinf/plant-multi/ (accessed on 21 June 2023).

### 4.3. Phylogenetic and Conserved Domain Analysis

To understand the evolutionary relationships among pitaya, *A. thaliana*, and the rice SWEET gene family. The identified SWEET protein sequences of pitaya, *A. thaliana*, and rice were merged, and a neighbor-joining (NJ) phylogenetic tree was constructed via the default parameters of MAGA11 [60]. The online website iTOL v4.0 (https://itol.embl.de/itol.cgi (accessed on 17 August 2023)) [61] and Adobe Illustrator software (version CS6) were subsequently used to annotate and modify the phylogenetic tree.

### 4.4. Analysis of Gene Structure and Conserved Motifs

TBtools software (version 1.113) was used to analyze the gene structure of each gene based on the pitaya genome annotation file (GFF3). The structural domain was subsequently predicted via the CD-search online tool of the NCBI database (http://www.ncbi.nlm.nih.gov/Structure/bwrpsb/bwrpsb.cgi (accessed on 22 June 2023)) with the default parameters [62]. We also used MEME (http://meme-suite.org/ (accessed on 22 June 2023)) online software to analyze the conserved motifs [63] of the SWEET proteins, thereby studying the differences among the HuSWEET family members. Finally, the above results were visualized via TBtools software (version 1.113) [64].

### 4.5. Analysis of Cis-Acting Elements

Gene IDs were obtained based on identification. The upstream 2000 bp nucleotide sequence of each gene was extracted from the genome annotation files (GFF3) via TBtools software (version 1.113), and the online analysis software PlantCARE (https://bioinformatics.psb.ugent.be/webtools/plantcare/html/ (accessed on 21 August 2023)) was used for promoter homeopathic element analysis [64].

### 4.6. Chromosomal Positioning, Gene Replication, and Collinearity Analysis

Chromosomal location information was obtained from the genome annotation file (DFF3), and chromosome location mapping was performed via TBtools software (version 1.113) [64]. Gene duplication events were subsequently analyzed via MCScanX, and the collinear relationships among the HuSWEET gene subfamily members of *A. thaliana*, rice, and pitaya were analyzed via TBtool software (version 1.113) [65].

### 4.7. Determination of Soluble Sugar Content

Pitaya seeds of ‘Baiyulong’ were used for tissue culture (1.5 mg/L 6-BA + 0.5 mg/L NAA + 0.4 mg/L IBA). The temperature of the culture room was 25 °C at day and 20 °C at night, and the light was provided 12 h per day, with a light intensity of 2000 lx. The seeds were germinated and grown in tissue culture flasks for four months. The pitayas obtained via subculture were divided into two groups. One group (low-temperature stress treatment group, denoted as T) was treated at 4 °C for 72 h in the dark [66]; the other group (control group, denoted as C) was incubated at room temperature in the dark. Three plants were treated in each group, and three biological replicates were set up for each treatment. A 0.5 g sample was cut and put into a mortar, where phosphate pH buffer and a small amount of quartz sand were added and ground until homogeneous. The homogenate and the residue were then diluted in a 100 mL volumetric flask, left at room temperature for 30–60 min (mixing once every 5 min), or centrifuged or filtered, and the residue was discarded. Next, sucrose standard solutions of different concentrations were reacted with phenol solution and concentrated sulfuric acid to generate compounds whose absorbances could be measured. By measuring the absorbance of standard solutions of different concentrations, a standard curve can be constructed, which can be used for subsequent quantitative analysis of samples. For sample measurement, 1.00 mL of the sample mixture was aspirated and processed according to the same steps used for standard curve preparation. After processing, the absorbance of the sample mixture was measured to determine the corresponding point on the standard curve; thus, the sugar content in the sample mixture was calculated [67]. For the low-temperature stress treatment group and the room temperature culture group, samples were collected at 0, 12, 24, 48, and 72 h, and three biological replicates were set up for each treatment.

### 4.8. Determination of the Activities of Catalase (CAT), Superoxide Dismutase (SOD), and Peroxidase (POD)

For the low-temperature stress treatment group and the room temperature culture group, samples were collected at 0, 12, 24, 48, and 72 h, and three biological replicates were set up for each treatment. Reagent solutions such as phosphate-buffered saline (PBS), methionine (Met), EDTA-Na_2_, riboflavin, hydrogen peroxide (H_2_O_2_), and nitroblue tetrazolium (NBT) were prepared. The leaves of the tissue-cultured plantlets were collected, ground in phosphate buffer in an ice bath, centrifuged, and the supernatant was obtained. The catalase (CAT) test kit (Baiolaibo Technology Co., Ltd., Beijing, China) was used to measure CAT enzyme activity, and the determination method followed the kit instructions. The enzyme mixture was added to the reaction mixture, and the change in absorbance at 240 nm was measured. CAT activity was calculated based on the change in absorbance [37]. The SOD enzyme activity was measured using a total superoxide dismutase (T-SOD) assay kit (Bohu Biotechnology Co., Ltd., Shanghai, China). Through chromogenic reactions, T-SOD activity was calculated by measuring the change in absorbance of each solution at 450 nm under specific conditions [37]. POD enzyme activity was measured using a peroxidase (POD) test kit (Renjie Biotechnology Co., Ltd., Shanghai, China). The enzyme mixture was added to the reaction mixture, and the change in absorbance at 470 nm was measured. The POD activity was calculated based on the change in absorbance [37].

### 4.9. ABA and MeJA Treatment

The histoculture medium (1.5 mg/L 6-BA + 0.5 mg/L NAA + 0.4 mg/L IBA) was added: 100 mM ABA or 100 mM MeJA. Three days after the addition of ABA and MeJA, the samples were taken at 0, 12, 24, 48, and 72 h at 4 °C, and three biological replicates were set for each treatment. Leaf samples were frozen in liquid nitrogen immediately after collection and stored at −80 °C for further experiments.

### 4.10. Growth Measurement

After low-temperature stress at 4 °C for 72 h, the cells were grown at room temperature for 30 d. A ruler was used to measure the distance from the shoot tip to the base of the plant. The plants should be kept vertical during measurement to ensure the accuracy of the measurement results. The fresh weight of the cultured tissue seedlings was determined with an electronic balance. Weight W = W30 − W0; root length L = L30 − L0; plant height H = H30 − H0.

### 4.11. Fluorescence Real-Time PCR Analysis

In the screening process, RT-qPCR analysis was performed at the time points with significant differences, mainly by measuring the soluble sugar content. To determine the significance of the differences, if there were significant differences, we further performed RT-qPCR analysis at different time points. Among the 28 genes, four DEGs were screened, among which three were upregulated and two downregulated; the remaining genes were either not expressed or their difference in expression was not significant.

Total RNA from each sample was extracted according to the instruction manual (DP441) of the RNAprepPur Polysaccharide and Polyphenol Plant Total RNA Extraction Kit (DP441) from Tiangen Biochemical (Beijing) Co., Ltd. (Beijing, China) The first cDNA strand was synthesized via reverse transcription via the RevertAid TM First Strand cDNA Synthesis Kit (Thermo Scientific, Waltham, MA USA). Specific primers for the *HuSWEET* genes were designed via the online website of NCBI (https://www.ncbi.nlm.nih.gov/ (accessed on 14 August 2023)) and synthesized by Shanghai Sangon Bioengineering Co., Ltd. (Shanghai, China) (Table 2). qPCR was performed using TB Green^®^ Premix Ex Taq™ (Beijing, China) reagent and a qTOWER3G real-time fluorescence quantitative system (Germany). The cDNA solution synthesized by total RNA extraction and reverse transcription was diluted 10-fold and used as a template for fluorescence quantitative PCR. The reaction system was 10 µL and contained 5 µL of TB Green^®^ Fast qPCR Mix (TaKaRa), 1 µL of cDNA template, 0.5 µL each of an upstream primer and a downstream primer, and 3 µL of sterile water. The reaction program was predenaturation at 95 °C for 3 min, followed by 40 cycles of 95 °C for 10 s, 60 °C for 20 s, and 72 °C for 15 s. The pitaya *HuActin* gene was used as an internal reference gene [68]. Each sample had three technical replicates. The relative expression level of each gene was calculated via the 2^−ΔΔCT^ method [46].

### 4.12. Protein Structure Prediction

BLAST alignment software (https://blast.ncbi.nlm.nih.gov/Blast.cgi/(accessed on 25 July 2024)) was employed to search for a known structural template with significant similarity to the target sequence. The protein structure prediction software SWISS-MODEL (https://swissmodel.expasy.org/(accessed on 25 July 2024)) was used to generate a preliminary three-dimensional structural model of the target protein. The specific steps were performed according to the method reported by Yang et al. [69].

### 4.13. Data Processing

Excel 2019 was used to organize the test data. SPSS 21.0 software was used for statistical analysis. Significance analysis was performed via one-way analysis of variance (ANOVA) and multiple comparison tests; bivariate correlation analysis was used to analyze the correlation between gene expression levels and enzyme activity. Origin2024 and Adobe Photoshop CS5 were used to create the images.

## 5. Conclusions

**Figure 12:**
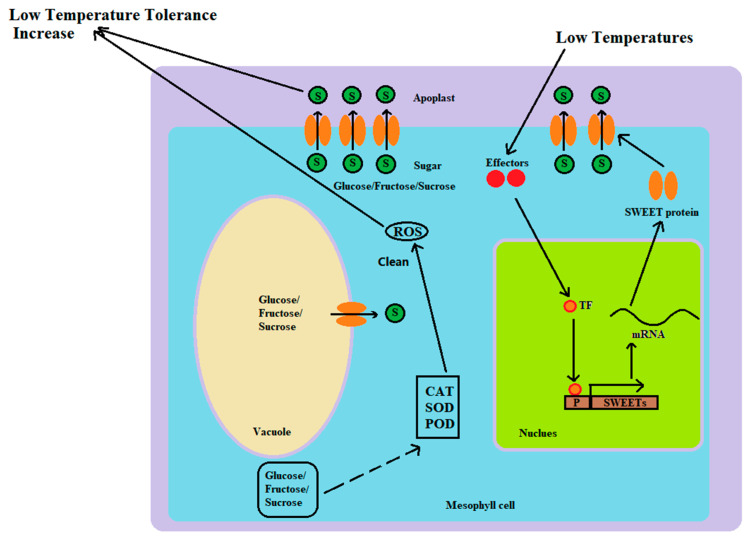
Molecular mechanisms involved in the response of SWEET gene family members to low-temperature stress. Note: P, promoter; TF, transcription factor; after cells are subjected to low-temperature stress, the expression levels of the SWEET gene family members increase to translate more proteins, which, in turn, results in the conversion of more total sugars (glucose/fructose/sucrose). On the one hand, the SWEET protein is delivered to the apoplast to improve the cold protection ability of pitaya cells; on the other hand, the increase in total sugar content in the cytoplasm indirectly increases the activity of the CAT, T-SOD, and POD enzymes, which scavenge the ROS produced in response to low-temperature stress and enhances the cold protection ability of pitaya cells. The solid lines represent direct action with no other process in between, while the dashed arrows illustrate indirect action with multiple action processes that have been omitted.

Twenty-eight members of the SWEET gene family were identified from the *H. undatus* genome. These family members can be divided into four subgroups. Members of this gene family presented some differences in the sequences of introns and exons, but their gene structure, especially their motifs, was relatively conserved. The promoter regions of most *HuSWEETs* have multiple stress- or hormone-related cis-elements. Owing to their specific function and expression mode, *SWEETs* play a crucial role in the cold resistance mechanism of pitaya. Within 72 h of low-temperature stress, *HuSWEET5* and *HuSWEET21* were upregulated first and then downregulated, *HuSWEET14* was always upregulated, and *HuSWEET12* was downregulated. These genes are involved in the response of pitaya to low-temperature stress. The exogenous hormones’, ABA and MeJA, application alleviated the damage caused by low-temperature stress on in vitro pitaya seedlings and significantly affected *HuSWEET5*, *HuSWEET12*, *HuSWEET14*, and *HuSWEET21* expression profiles. The possible molecular mechanism (see Figure 12) of the function of the SWEET gene family members of pitaya is to improve the cold tolerance of pitaya plants by increasing the total sugars in the apoplast and cytoplasm and indirectly increasing the activities of the CAT, T-SOD, and POD enzymes to scavenge ROS. This study provides a theoretical basis for breeding cold-resistant varieties of pitaya. It provides a foundation for improving the cold tolerance of pitaya through genetic engineering technology in practice.

## Figures and Tables

**Table 1:** Analysis of the physical and chemical properties of the HuSWEET gene family members in *H. undatus*.

Sequence ID	Gene Name	Amino Acid	MW(kDa)	pI	GRAVY	TMHs	Location
HU01G01645.1	HuSWEET1	294	32.44	8.85	0.486	7	Plasmid IMP
HU01G02211.1	HuSWEET2	309	34.74	9.45	0.593	7	Plasmid IMP
HU01G02212.1	HuSWEET3	309	34.74	9.45	0.593	7	Plasmid IMP
HU01G02213.1	HuSWEET4	293	33.12	9.12	0.34	7	Plasmid IMP
HU01G02214.1	HuSWEET5	257	28.99	8.68	0.311	5	Plasmid IMP
HU02G01006.1	HuSWEET6	333	37.57	5.71	0.368	7	Plasmid IMP
HU02G01007.1	HuSWEET7	342	38.39	8.15	0.356	7	Plasmid IMP
HU02G01008.1	HuSWEET8	182	20.49	8.63	0.221	4	Plasmid IMP
HU02G02490.1	HuSWEET9	258	28.64	9.69	0.583	7	Plasmid IMP
HU03G01299.1	HuSWEET10	257	28.33	9.47	0.557	7	Plasmid IMP
HU04G00350.1	HuSWEET11	211	23.10	8.38	0.647	6	Vacuolar IMP
HU04G00351.1	HuSWEET12	256	27.86	9.28	0.788	7	Plasmid IMP
HU04G01094.1	HuSWEET13	135	14.96	9.05	0.939	5	Endoplasmic reticulum IMP
HU04G01322.1	HuSWEET14	301	34.13	9.14	0.772	7	Plasmid IMP
HU04G01323.1	HuSWEET15	318	35.53	8.74	0.501	7	Plasmid IMP
HU04G01325.1	HuSWEET16	315	34.87	8.96	0.534	7	Vacuolar IMP
HU04G01326.1	HuSWEET17	335	37.37	7.02	0.542	7	Plasmid IMP
HU04G01327.1	HuSWEET18	289	32.14	8.32	0.391	6	Plasmid IMP
HU04G02123.1	HuSWEET19	192	21.82	9.59	0.563	6	Plasmid IMP
HU04G02228.1	HuSWEET20	211	23.10	8.71	0.64	6	Vacuolar IMP
HU04G02229.1	HuSWEET21	256	27.83	9.24	0.783	7	Plasmid IMP
HU07G00905.1	HuSWEET22	257	28.01	9.32	0.477	7	Plasmid IMP
HU07G01376.1	HuSWEET23	332	37.39	8.18	0.387	7	Plasmid IMP
HU08G01549.1	HuSWEET24	260	28.57	9.32	0.635	7	Plasmid IMP
HU08G01881.1	HuSWEET25	235	26.30	9.17	0.87	7	Vacuolar IMP
HU09G00626.1	HuSWEET26	243	27.03	9.32	0.53	7	Cytoplasmic IMP
HU09G00864.1	HuSWEET27	258	28.64	9.69	0.583	7	Plasmid IMP
HU10G00025.1	HuSWEET28	216	24.39	7.62	0.479	6	Vacuolar IMP

Note: IMP, integral membrane protein.

**Table 2:** Primer sequences used for *HuSWEET* gene expression analysis.

Primer Name	Primer Sequence (5′–3′)	Product Size	Tm	Application
HuSWEET1	AAGCTACCAGAGCAGGTCCA	168	60.01	qRT-PCR
HuSWEET1	GACAATTTTGCCGTCCTCAT		60.04	qRT-PCR
HuSWEET2	CTTGGATGGATTTGCCTCAT	166	59.91	qRT-PCR
HuSWEET2	CGAATCACTTGCCTCTGTCA		59.98	qRT-PCR
HuSWEET3	CTTGGATGGATTTGCCTCAT	166	59.89	qRT-PCR
HuSWEET3	CGAATCACTTGCCTCTGTCA		59.98	qRT-PCR
HuSWEET4	CTCTTTGGGATGGCACAAAT	181	59.93	qRT-PCR
HuSWEET4	GCTGAGTTGGCATCGTGATA		59.83	qRT-PCR
HuSWEET5	CTCTTTGGGATGGCACAAAT	178	59.93	qRT-PCR
HuSWEET5	CTTGCTGAGGTGGCATCATA		59.82	qRT-PCR
HuSWEET6	AAGCCGAGCTCTGCATAAAA	168	60.12	qRT-PCR
HuSWEET6	TTACCGTCTCCGTCTCCATC		60.07	qRT-PCR
HuSWEET7	ACGGTTTCCTCATTCGTGAC	220	59.97	qRT-PCR
HuSWEET7	AGGTCGTCTTGGGGTTTCTT		59.97	qRT-PCR
HuSWEET8	ACCGTTATGGTGAGGCTGTC	154	60.00	qRT-PCR
HuSWEET8	GAACTCCACGCTCTTCGTTC		60.00	qRT-PCR
HuSWEET9	TCTTTTGCCGACTCCCTAGA	150	59.95	qRT-PCR
HuSWEET9	GCCAACTGAGAGCCAAAAAG		59.99	qRT-PCR
HuSWEET10	GGGGGCTGGAAAGATAAAAC	179	59.78	qRT-PCR
HuSWEET10	GCTGGCTCTTTTGTGCTACC		60.02	qRT-PCR
HuSWEET11	TGGGTTTTCCAATCTGCATT	184	60.31	qRT-PCR
HuSWEET11	GCTGCTAGCTGCTCTTTGGT		59.93	qRT-PCR
HuSWEET12	GCGACAAGCCAGGTGATACT	159	60.29	qRT-PCR
HuSWEET12	GATCGGGTTCTCATCGTTGT		59.93	qRT-PCR
HuSWEET13	GCTCCTTCGACTGCCTTCTA	171	59.72	qRT-PCR
HuSWEET13	CACAGCTCAGAAACCCAACA		59.87	qRT-PCR
HuSWEET14	TCCTTCCCACTTTAGCGTGT	171	59.73	qRT-PCR
HuSWEET14	GCAACCACATAGGGAATGCT		59.96	qRT-PCR
HuSWEET15	AATGTGGTGGGGTTCATGTT	151	59.95	qRT-PCR
HuSWEET15	TGTTTGGCTGTATTCCCACA		59.96	qRT-PCR
HuSWEET16	AGAGCAGGTCCAGGTCTCAA	154	59.99	qRT-PCR
HuSWEET16	TTTGCCCTCCTCATTTTCAG		60.18	qRT-PCR
HuSWEET17	CAACCCTCAAGCTCCTTCTG	151	59.98	qRT-PCR
HuSWEET17	CTAAGAGGTGCCGCAAAGAC		60.02	qRT-PCR
HuSWEET18	CCAAGTGAGTGTTGCTCCAA	173	59.87	qRT-PCR
HuSWEET18	GCAGGTCGAAAGAGAGATGC		60.10	qRT-PCR
HuSWEET19	GCTTTGTGTGTTGCCTTCAA	166	59.89	qRT-PCR
HuSWEET19	GCATCCCCACAAAATCATTC		60.14	qRT-PCR
HuSWEET20	TGGGCACTCTATGGCTTACC	179	60.10	qRT-PCR
HuSWEET20	ATGCCAACGGCAAGTATCTC		60.10	qRT-PCR
HuSWEET21	GCGACAAGCCAGGTGATACT	159	60.29	qRT-PCR
HuSWEET21	GATCGGGTTCTCATCGTTGT		59.93	qRT-PCR
HuSWEET22	TACCAAATGGGTTTGGGTGT	176	59.95	qRT-PCR
HuSWEET22	ACCATTAGGCTTGGGCTTTT		59.97	qRT-PCR
HuSWEET23	TTGGGTTGGGATATTTGCAT	201	60.02	qRT-PCR
HuSWEET23	CGATGGAGTTGATGGTGATG		59.92	qRT-PCR
HuSWEET24	GGTTTTCTTGGCACCAGTGT	198	60.01	qRT-PCR
HuSWEET24	CAATGCACTGAAGAGGGACA		59.83	qRT-PCR
HuSWEET25	ACACGACGAAGAGGAGAGGA	154	59.99	qRT-PCR
HuSWEET25	TTTAAACCCTGCGAGAATGG		60.07	qRT-PCR
HuSWEET26	TCACCCTTCTGGTTTTCCAC	158	59.94	qRT-PCR
HuSWEET26	TTCCAACAATGCACCAAAAA		59.95	qRT-PCR
HuSWEET27	TCTTTTGCCGACTCCCTAGA	150	59.95	qRT-PCR
HuSWEET27	GCCAACTGAGAGCCAAAAAG		59.99	qRT-PCR
HuSWEET28	TTGCACGTTGCAGGTAAAAG	166	59.91	qRT-PCR
HuSWEET28	GGGGAGATGGGAGTTTGAAT		60.13	qRT-PCR

## Data Availability

All the data generated or analyzed in this study are included in this article.

## Supplementary Materials

The following supporting information can be downloaded at: https://www.mdpi.com/article/10.3390/plants13213092/s1, Table S1: *HuSWEETs*’ expression after 6 h for 4 °C treatment and at room temperature.
